# Data reuse in global health: perspectives from actors in policy, funding and research

**DOI:** 10.1136/bmjgh-2025-021974

**Published:** 2026-03-04

**Authors:** Naomi Waithira, Evelyne Kestelyn, Mavuto Mukaka, Dung Nguyen Thi Phuong, Keitcheya Chotthanawathit, Hoa Nguyen Thanh, Rachel Odhiambo, Jennifer Van Nuil, Phaik Yeong Cheah

**Affiliations:** 1 Centre for Tropical Medicine and Global Health, University of Oxford Nuffield Department of Medicine, Oxford, UK; 2 Mahidol Oxford Tropical Medicine Research Unit, Bangkok, Thailand; 3 Oxford University Clinical Research Unit Vietnam, Ho Chi Minh City, Viet Nam

**Keywords:** Global Health, Qualitative study, Decision Making, Health policies and all other topics, Diagnostics and tools

## Abstract

**Background:**

Data-sharing mandates from funders and journals have increased in recent years, but little is known about how shared data are used. Existing research has focused on access frameworks, with less attention to conditions that enable or hinder subsequent analyses and their impact on science and policy.

**Methods:**

We conducted semi-structured interviews with 22 key informants with experience using clinical research data. Participants included researchers, policy makers and senior staff from funding and pharmaceutical organisations. Interviews explored motivations, ethical and practical challenges, and enabling conditions for reuse. Data were analysed thematically using a combination of deductive and inductive coding. Reporting follows the Consolidated criteria for Reporting Qualitative research framework.

**Results:**

Secondary data analyses have, in a few documented cases, shaped clinical guidelines and policy in low- and middle-income countries (LMICs). Individual participant data meta-analyses informed WHO recommendations for maternal and child health interventions, and analyses of COVID-19 data guided decisions at national and subnational levels in several countries. However, such cases remain uncommon. Secondary data users reported that shared data were seldom ready for analysis owing to incomplete metadata and under-resourced data curation. In academia, secondary analyses were driven by the potential for publication rather than health impact. Mistrust, particularly where data contributors feared reputational harm or exploitation, resulted in underutilisation of valuable data as analysts relied on a limited set of well-known or easily accessible datasets. This risks selection bias and limits the evidence base, especially for under-represented groups.

**Conclusions:**

Mandating data sharing alone is insufficient to deliver impact in LMICs. Policies must be coupled with resourcing for data curation, efforts to avail machine-actionable metadata and incentives for impact-driven analyses. Equally critical is trust, built through recognition of contributors and equitable, transparent benefit-sharing between analysts and data generators.

**WHAT IS ALREADY KNOWN ON THIS TOPIC**

- Data scarcity continues to constrain conventional and artificial intelligence-driven analyses despite increased mandates for open science.

**WHAT THIS STUDY ADDS**

- We highlight instances where analysis of shared individual-patient data enabled rapid outbreak response and produced evidence for neglected groups such as children and pregnant women.
- However, most research data still remain underused due to technical (incomplete metadata, weak documentation and curation), structural (publication-oriented incentives) and social barriers (mistrust due to fears of reputational harm and extractive practices).

**HOW THIS STUDY MIGHT AFFECT RESEARCH, PRACTICE OR POLICY**

- Data sharing mandates must be coupled with investment in curation of existing datasets, clear attribution frameworks and institutional structures that value the effort of data generation and secondary analyses.

## Introduction

 Reuse of health data to generate new evidence, replicate findings or inform policy is widely promoted but remains poorly characterised and understood. Funders and journals increasingly require that individual participant data (IPD) from publicly funded research be shared via repositories or collaborative platforms. These policies are based on the view that data sharing enhances transparency, accelerates discovery and maximises the scientific value of research investments.^1^,^4^

Despite this shift in policy, far less attention has been paid to whether and how shared datasets are actually used. Most literature focuses on frameworks and incentives for making data accessible, often under the assumption that reuse will naturally follow. However, evidence suggests that this is not the case. For data generated in high-income settings, practical and ethical barriers have been documented and include legal and governance hurdles, data quality issues and gaps in analytical skills, which often prevent researchers from accessing or analysing shared datasets.

In order to promote the reuse of shared data, initiatives such as ‘datathons’ and data challenges have emerged. These initiatives bring together multidisciplinary teams to generate new findings or tools from secondary analysis within defined timeframes. Some offer financial incentives to encourage participation. While promising, these efforts are short-term and require sustained investment and closer alignment with local research priorities, both of which should be informed by evidence.^5 6^

Much of the available evidence reflects the views of data producers, repository managers and funders, and relates to data generated from high-income settings.^7^,^12^ Most global health research relies on low- and middle-income countries (LMICs). Challenges with reusing these data are likely to be more acute as infrastructure and institutional support remain limited.^13^,^17^ Yet the perspectives of those working with data from these settings remain underexplored.

We aim to address this evidence gap by investigating the experiences of individuals who have engaged in secondary analyses of health data generated in LMIC settings. The study addresses three questions: how do researchers access and work with shared health data? what obstacles limit their ability to carry out secondary analyses? and what forms of support or change are needed to improve the utility and impact of shared datasets?

## Methods

We conducted a mixed-methods study comprising a cross-sectional survey and in-depth interviews. This paper reports the qualitative findings that explored stakeholders’ experiences and perspectives. The study protocol is published and provides further details on the study design and procedures.^18^

We used purposive and snowball sampling to recruit individuals with demonstrated experience in secondary analysis of health data generated in clinical studies conducted in LMICs. Initial participants were identified through a structured PubMed search (search strategy provided in online supplemental appendix 1), supplemented by institutional reports and public professional profiles. Through purposive sampling, we sought variation in institutional role, disciplinary background and geographic location. Additional participants were identified by referral.

We invited 32 individuals, of whom 21 responded and all consented. In total, 21 interviews were conducted with 22 key informants, as one interview included two individuals. The sample comprised researchers, policy advisors and senior staff from regulatory, funding and public health agencies. Two-thirds were based in LMICs, and 59% identified as women. Interviewees represented diverse fields including clinical research, epidemiology, artificial intelligence, statistical modelling and health policy (table 1).

**Table 1:** Characteristics of key informants (N=22)

Category	Subcategory	n (%)
Sector*	Academic research	10 (45.5)
	Regulator/policymaker	5 (22.7)
	Pharmaceutical/industry	2 (9.1)
	Funding	2 (9.1)
	Non-governmental agency	4 (18.2)
Employer location (by income level)	High-income country (HIC)	8 (36.4)
	Low- and middle-income country (LMIC)	14 (63.6)
Employer location (by region)	Asia	8 (36.4)
	Africa	4 (18.2)
	Americas	5 (22.7)
	Europe/UK	5 (22.7)
Gender	Man	9 (40.9)
	Woman	13 (59.1)
Expertise*	Epidemiologist	3 (13.6)
	Clinician	2 (9.1)
	Health system/management	5 (22.7)
	Algorithm developer (including AI)	3 (13.6)
	Statistician	3 (13.6)
	Computational modeller	5 (22.7)
	Clinical researcher	4 (18.2)
Area of study*	Infectious diseases	10 (45.5)
	Antimicrobial resistance	12 (54.5)
	Chronic illnesses	12 (54.5)

* Categories are not mutually exclusive.

AI, artificial intelligence.

Semi-structured interviews lasting 45–60 min were conducted virtually between March 2021 and April 2024, following an interview guide (online supplemental appendix 2), developed from a prior literature review. Topics included motivations for reuse, processes of data discovery and access, ethical and analytical challenges, and recommendations for improving the utility of shared datasets. 18 interviews were conducted in English and three in Vietnamese, with the latter transcribed and translated by bilingual staff (DH, HN).

All interviews were audio-recorded with consent and transcribed verbatim. Field notes were taken during the interviews and during team coding meetings. Data collection continued until thematic saturation was reached, as independently assessed by four members of the research team (NW, PYC, EK and JVN). The research team comprised six researchers with multi-disciplinary backgrounds, including medical anthropology, clinical research, ethics and data science. Interviewers (NW, KC, HN) had prior training in qualitative data collection and analysis and had no personal relationships with participants. Reflexive discussions were held prior to data collection and during analysis to identify and document potential biases and positionality in relation to the research topic. The study team members have had professional experience in clinical research and data sharing initiatives and therefore may have shaped the interpretation of the findings, particularly on operational and governance aspects.

We conducted thematic analysis using a hybrid deductive-inductive approach. Transcripts were coded in NVivo (V.14). An initial coding framework was developed based on a prior literature review and results of the quantitative survey conducted as part of this mixed-methods study.^19^ The framework was iteratively refined as new codes emerged during analysis. 20% of transcripts were independently coded by two researchers (KC and NW). Discrepancies were discussed and the coding framework was refined. Final codes were reviewed by the research team to ensure consistency and thematic coherence. Reporting follows the Consolidated criteria for Reporting Qualitative research, detailed in online supplemental appendix 3.

## Results

The findings are organised in four thematic domains: (1) patterns of data reuse; (2) operational barriers to data reuse; (3) the role of ownership and trust and (4) shifting norms and impact of evidence of data reuse.

### Patterns of data reuse

Across sectors, participants reported primarily relying on data from observational studies and routine health surveys (eg, Demographic and Health Surveys (DHS), UNICEF’s Multiple Indicator Cluster (MIC) surveys).

There was a strong preference for publicly published datasets attributed to their ease of access. In contrast, data from randomised clinical trials (RCTs) and electronic health records were underused due to regulatory restrictions and complex approval processes. The time required for approval made the data unusable in practice. “I was not able to work with the RCT dataset because it took too much time to get clearance” (Analyst at regulatory agency, USA)

Usage patterns varied by data type. Observational data, health records and wearable devices data were typically used for epidemiological trend analyses, exploratory research and predictive modelling. RCT data were predominantly used in IPD meta-analyses, mostly assessing drug safety and efficacy.

Although peer-reviewed publications were the primary goal for most analysts, in practice, grey literature such as policy briefs, technical reports and conference materials was most commonly produced. Some associated this with prolonged peer-review timelines and data access restrictions that necessitated alternative dissemination channels, particularly for findings intended to inform timely decision-making. However, within disciplines that prioritise journal-based metrics, grey literature carries less academic recognition, thus constraining dissemination choices. As a result, analysts described a tension between institutional expectations and utility. Although some analyses were completed to a high standard, they were not disseminated, resulting in wasted analytical effort and a gap in the cumulative scientific evidence.

### Operational barriers to data reuse

Participants described practical constraints that hindered efforts to run secondary analyses.

#### Data discovery

Locating relevant datasets was reported as a major challenge driven by poor cataloguing, inconsistent metadata and institutional silos. This was particularly acute for observational studies, which typically lack the formal registration required for clinical trials. Existing repositories lacked functionality to allow users to determine the relevance of datasets for their needs and publication bias skewed availability towards studies with positive or statistically significant outcomes. To improve data discovery, participants advocated the development of publicly accessible data catalogues, use of standardised metadata and persistent identifiers (eg, digital object identifiers (DOIs) for datasets and ORCID IDs for contributors).

#### Methodological incompatibility

Even when datasets could be found, methodological limitations frequently prevented meaningful secondary analyses. Datasets collected for specific research objectives often lacked breadth or granularity for secondary use. Inconsistencies in case definitions, diagnostic criteria and measurement instruments further posed challenges for comparative or pooled analyses. As one participant contributing to a Cochrane review noted, “Malaria infection outcomes were measured differently, by microscopy, RDT and PCR. You can’t just combine those in one bucket” (Analyst, USA).

To address these limitations, analysts applied techniques such as sensitivity analyses and Bayesian modelling and explicitly acknowledged data limitations within their findings. As one analyst noted: *“*Our definition of full vaccination was seven doses, but the vaccination percentage (reported) in the Multiple Indicator Cluster Surveys was different. In our report, we specified our formula and explained how our calculations differed from other published results” (Infectious disease researcher, Vietnam).

Several called for wider adoption of guidelines in study design and reporting (such as Standard Protocol Items: Recommendations for Interventional Trials, Strengthening the Reporting of Observational Studies in Epidemiology, Consolidated Standards of Reporting Trials) and use of validated data collection instruments to improve cross-study comparability.

#### Data curation and documentation practices

Data curation was described as under-resourced by all participants. Many datasets were provided with little or no documentation. Inconsistent formats and heterogeneity in study methodology necessitated substantial data cleaning and harmonisation before analysis could begin. Curation required significant time and expertise, yet was rarely funded or formally recognised. A geospatial analyst working in Laos, Myanmar and Thailand described spending ‘80% of their time curating data before they could start analysing it’, citing repeated efforts to resolve inconsistencies.

Participants called for funders to not only mandate data sharing but also allocate resources to curate existing datasets, and to formally support stewardship in new grants.

To fill documentation gaps, researchers frequently relied on informal engagement with data producers to contextualise data and interpret findings.

We reach out to investigators as often times contextual information isn’t published. (Infectious disease researcher, USA)

Such engagement, while critical, was time-consuming, dependent on goodwill and often inaccessible to those without established relationships. This informal workaround also remained unrecognised in governance frameworks.

### Ownership and trust

#### Data ownership

In academic settings, data were frequently treated as an intellectual asset tied to career progression and publication credit. Participants noted that the substantial time and effort required to secure funding and to generate primary data reinforced a sense of responsibility for how those data were subsequently used, that in some instances, led to extended embargo periods or restricted access. This was described as more of a concern about how findings might be interpreted once data were removed from their original study context, particularly where documentation and metadata were limited, than an attempt at control. From a funder’s perspective, the concerns were acknowledged but weighed against wider responsibilities for scientific value and public accountability. As one funder from the USA noted:

The biggest problem is not informed consent or whether the data sit in a database. The biggest problem is people don’t want to share because they think they own it. Once they’ve published and squeezed out one more paper, they should just let it go. Move on to the next study and let others take a look. As a public funder, it’s about the science, not how many papers you can add to your CV.

Industry informants, on the other hand, described data as a commercial asset. While openness was increasing, access remained shaped by proprietary concerns and regulatory compliance:

We use (industry) data and we buy it, it is now proprietary to our organization. (Health economist at a pharmaceutical company, India)

#### Trust

Trust emerged as a key determinant of access. Past experiences, perceptions of unreciprocated data sharing and limited recognition in analysis and publication caused LMIC data providers to be cautious of data requests. Some participants from high-income countries reported difficulties accessing data from LMIC due to perceptions of inequity, where better-resourced institutions were viewed as extracting value without reciprocation.

People don’t want to give to those who already have more than enough. (Coordinator, IPD meta-analysis, UK)

Concerns were raised about the benefits to communities from which data were derived, particularly when reuse occurred at scale:

They want to say they used data from millions of LMIC patients, but did they think about what comes back to us? (Senior clinical researcher, Thailand)

In antimicrobial resistance research, mistrust was aggravated by concerns of political or reputational consequences arising from misinterpreted findings. Similarly, in the context of clinical trials, fears of participant harm and regulatory non-compliance resulted in defensive governance structures and cautious access controls. While these measures were intended as safeguards, they often introduced bureaucratic delays that impended timely analysis:

Negotiating a data transfer agreement can take time. Some members of the review board have negative sentiments toward foreign institutions, so they need a bit more convincing. The intention is positive, but the implementation isn’t straightforward and presents another challenge rather than an enabler. (Medical doctor/research governance expert, Indonesia)

However, while formal governance processes proved slow in many instances, access decisions were often linked to existing professional relationships. As one informant reflected, access to the same dataset could vary markedly on the basis of who was asking rather than formal criteria: “Why is it that with the same set of data, one person gets it, the other doesn’t? If I didn’t have good personal relationships (with the data contributors), I would never have been able to access the data. I definitely have to admit that” (Senior epidemiologist, Vietnam).

Across these accounts, it emerges that trust cannot be legislated, but must be built through reciprocal, transparent partnerships that recognise data contributors as co-producers of knowledge.

### Shifting norms and impact evidence

Despite systemic challenges, participants pointed to instances where the scientific and policy value of data sharing was realised. Pooling data across geographies enabled research in historically excluded populations such as children and pregnant women.

We recently completed a study in over 11,000 children on malnutrition and treatment outcomes. Without pooled IPD from across Africa, we wouldn’t have reached that conclusion. (Analyst, South Africa)

In some cases, findings from secondary analyses directly informed treatment guidelines, national policy decisions or supported real-time planning at the hospital level, such as triaging treatment protocols or identifying resource gaps during outbreaks. As one participant reflected:

During my PhD, many years ago, I developed a model about how to better control or prevent hepatitis B infections. Yes, we submitted it to a mathematical journal, but after discussing with the Ministry of Health, the strategy implemented at national level was based on the one we presented. For me, just finishing my PhD, I was very, very happy because I saw mathematical modelling really help solve a real problem. (Mathematical modeller, West Africa)

Beyond policy, participants described how engagement in data reuse projects contributed to career progression through new collaborations, expanded skillsets and visibility in global networks. In these instances, success hinged on deliberate collaborations with data contributors and analysts, and institutional support. Collaborative models of analysis where data contributors co-design research questions, interpret findings and share authorship were cited as exemplars of rigorous and equitable practice.

The value comes when everyone helps shape the question and the analysis. That collective intellect is stronger than any one mind. (Group head and coordinator, IPD meta-analyses, South Africa)

Informants in senior management and funding roles described efforts to encourage data stewardship and secondary analysis, mostly within large consortia or funder-driven initiatives. These included the establishment of dedicated data repositories and grant schemes to support data curation and reuse. However, such initiatives are few and limited to specific disease areas.

At the institutional and journal level, participants pointed to data-sharing policies that set expectations around co-authorship for data contributors, requirements for assigning DOIs to shared datasets and performance review processes in which grey literature outputs were considered. These policies were described as variably implemented, which limited their influence on routine academic practice.

Analysts, particularly those in computational modelling, described changing community practices such as sharing analytical code to support transparency and reproducibility.

Practices are changing in some settings, but their long-term integration into routine research remains uncertain.

## Discussion

In this study, we examine secondary analysis of health data generated in LMIC contexts, and whether data sharing initiatives deliver meaningful scientific and population health benefit. Our findings show that reuse is constrained by difficulties in locating relevant datasets, insufficient information to interpret them appropriately and access barriers arising from lack of trust. Yet despite these constraints, secondary analyses have in some instances delivered demonstrable impact.

### Patterns and motivations of data reuse

IPD meta-analyses have generated policy-shaping evidence for pregnant women and children, populations often excluded from primary research. For example, a meta-analysis including 7072 children from multiple studies directly informed WHO treatment guidelines in Africa and Asia.^20 21^ A pooled analysis of over 11 000 participants showed that acute malnutrition significantly increased the risk of treatment failure,^22^ while another provided critical evidence on drug safety in pregnancy.^23^ However, IPD meta-analyses remain relatively uncommon in LMIC contexts, due to logistical complexity, restrictive data access procedures and significant requirements for data harmonisation.^24^ Data from RCTs, despite their potential to inform high quality synthesis, remain underused. This has been reported in other settings, but the implications are more acute in LMIC where trial data are already few.^25^

Routinely collected survey data have been repurposed to support real-time public health responses, including during the COVID-19 pandemic.^26^ Many respondents in our study reported routinely using openly accessible datasets, such as DHS and UNICEF MIC surveys for exploratory analyses, modelling studies and rapid assessments due to their ease of access and usability, rather than their methodological rigour or representativeness. Scientifically, overreliance on a limited subset of datasets risks introducing analytical biases.^27^ Sustainability is also at risk, as demonstrated by the recent funding shift that threatened the DHS dataset.^28^ There is an urgent need to diversify the datasets available for secondary analyses.

### Operational barriers to data reuse

Accessibility does not equate to usability. Technical limitations such as insufficient documentation, inconsistent data formats and missing metadata continue to limit secondary use. The FAIR guidelines propose solutions to these challenges, but their implementation is yet to pick up. Additionally, many available datasets suffer methodological limitations such as inadequate sample sizes or study design flaws that limit generalisability. Due to these shortcomings, appraisal of datasets for analytic suitability and harmonisation of variables across multiple datasets is essential. Analysts often rely on direct communication with data contributors to fill contextual and documentation gaps. While this may allow immediate analysis to proceed, it is inefficient and inequitable. Systematic documentation of datasets with standardised and machine-actionable metadata is a necessity for data discovery and interpretation as described in the EQUATOR Network’s 2024 recommendations.^29^ Our findings suggest that in LMIC-focused academic and public-sector work, funding and governance arrangements rarely state how curation and documentation should be resourced. As such, curation is implicitly a responsibility for data producers or host institutions, while secondary users benefit from prepared datasets at little or no cost.^30^ By contrast, industry datasets are treated as commercial products, with access usually governed through fees or licences that can be used to support curation. This imbalance has predictable effects on sustainability, fairness and incentives and helps to explain why, despite formal data-sharing policies, many datasets are only partly prepared for reuse. It is essential that data curation activities receive appropriate resourcing and explicit recognition within authorship guidelines and academic reward systems. Practical models include budgeting curation within primary grants, funding institutional curation services and allowing limited cost-recovery from secondary users.

Even when datasets are accessible and well documented, subsequent use is often guided by the incentives embedded within academic institutions rather than scientific or public health priorities. There is greater reward for publishing in peer-reviewed journals, which, as our informants noted, can take precedence over producing outputs such as policy briefs or dashboards that may offer greater real-world impact. This presents a paradox: while data sharing is intended to advance health and science, current incentives often push researchers to prioritise academic visibility over what delivers most benefit in public interest.

### Ownership and trust

While institutional incentives shape how data are reused, our findings indicate that trust is an even more critical factor in LMIC settings. Trust cannot be legislated, and in the absence of enforceable attribution norms or meaningful involvement in subsequent analyses, data producers often lack confidence that secondary use will fairly reflect their contributions or safeguard their reputations.^31^ In politically sensitive contexts, such as infectious disease outbreaks, concerns about stigma or reputational harm can prompt authorities to restrict access, as seen during the COVID-19 pandemic in South Africa. However, our study also shows that collaborative analyses build trust and deliver value to all parties, when data contributors are actively engaged in shaping research questions and analytic frameworks. Making these positive examples more visible could help reframe data sharing as a genuine opportunity rather than a bureaucratic obligation.

### Recommendations for policy and practice

Based on these findings, we propose the following recommendations to address existing barriers and further the impact of secondary analyses of data generated in LMIC contexts:

- Improve the findability of datasets:
  - Establish field-specific data annotation standards through collaborative working groups.
  - Create and sustain shared metadata repositories managed by multi-institutional consortia.
  - Funders and research institutions to provide formal support for metadata development and stewardship.
- Institutionalise and resource data curation:
  - Establish and resource central data curation units or support services within institutions to provide technical and administrative assistance for dataset preparation and reuse.
  - Develop and disseminate open-source toolkits and workflow templates based on established data standards and integrate their use in real-world studies within capacity building programmes.
- Broaden recognition and reward systems:
  - Revise promotion, tenure and grant evaluation criteria to explicitly recognise outputs such as technical reports, policy briefs and open datasets.
  - Encourage journals and funders to include grey literature citations as legitimate research contributions in reporting and review processes.
- Equitable analytical partnerships:
  - Co-development of analysis plans between secondary users and data contributors to integrate local context and expertise.
  - Adopt clear authorship and attribution guidelines where data contributors specify expectations for their role and consultation expectations upfront.

### Strengths and limitations

This study focused on the practical realities of data reuse, a shift from current literature that largely explores philosophical and theoretical concepts of data sharing. We draw on the experiences of individuals with direct involvement in secondary analyses of LMIC datasets to provide a grounded view of how data sharing is and could advance science and better health. The study sample captures views from diverse roles, sectors and regions. While not exhaustive, this diversity strengthens the relevance of the findings across settings.

As with any qualitative study, the findings reflect the perspectives of those interviewed and may not capture the full range of experiences elsewhere. Recruitment through professional networks and public outputs may have favoured those with greater visibility. Participants worked primarily with quantitative data, which may limit the relevance of these findings to qualitative research contexts.

## Conclusion

If data sharing is to fulfil its promise in global health, data reuse must be treated as an integral part of the research process, not its by-product. This study shows that reuse is most effective when it is actively supported: through investment in curation of existing datasets, clear attribution frameworks and institutional structures that value the labour of data preparation and secondary analysis. The persistence of informal workarounds and uneven access to data points to a system not yet equipped to deliver on the expectations it sets. Realising the full value of shared data will require moving beyond data sharing mandates to strengthening systems that make reuse both possible and worthwhile for those who generate data and for those who use it.

## Supplementary material

10.1136/bmjgh-2025-021974 online supplemental file 1

10.1136/bmjgh-2025-021974 online supplemental file 2

10.1136/bmjgh-2025-021974 online supplemental file 3

10.1136/bmjgh-2025-021974 online supplemental file 4

## Data Availability

Data are available upon reasonable request.
