# Supplementary material for: Data reuse in global health: perspectives from actors in policy, funding and research
Source: BMJ Glob Health. 2026 Mar 4;11(3):e021974. doi: 10.1136/bmjgh-2025-021974 (PMC12970136; doi:10.1136/bmjgh-2025-021974)

**Search strategies**

| **Source** | **Search Date** | **Full Search String** | **Filters** | **Sort Order** | **Results (n)** |
| --- | --- | --- | --- | --- | --- |
| PubMed (MEDLINE) | May 2025 | (("data reuse"[All Fields] OR "reuse of data"[All Fields] OR "secondary data analysis"[All Fields] OR "secondary analysis"[All Fields]) AND ("health data"[All Fields] OR "clinical trial data"[All Fields] OR "patient data"[All Fields]) AND ("low and middle income countries"[All Fields] OR "LMIC"[All Fields] OR "global health"[All Fields] OR "low resource"[All Fields] OR "LRS"[All Fields] OR "resource-poor setting"[All Fields] OR "resource-poor settings"[All Fields] OR "less developed countries"[All Fields] OR "emerging economies"[All Fields] OR "under-resourced countries"[All Fields] OR "resource-constrained settings"[All Fields] OR "low-GDP countries"[All Fields] OR "economically developing nations"[All Fields] OR "low- and middle-resource countries"[All Fields] OR "global south"[All Fields]) ) AND (("1900"[Date - Publication] : "2021"[Date - Publication])) | Publication date: before 2022 | By Publication Date | n = 17 |
| SCOPUS (Elsevier) | May 2025 | TITLE-ABS-KEY("data reuse" OR "reuse of data" OR "secondary data analysis" OR "secondary analysis")  AND TITLE-ABS-KEY("health data" OR "clinical trial data" OR "patient data")  AND TITLE-ABS-KEY("low and middle income countries" OR LMIC OR "global health" OR "low resource" OR LRS  OR "resource-poor setting" OR "resource-poor settings" OR "less developed countries" OR "emerging economies"  OR "under-resourced countries" OR "resource-constrained settings" OR "low-GDP countries"  OR "economically developing nations" OR "low- and middle-resource countries" OR "global south")  AND PUBYEAR < 2022 | Publication date: before 2022 | By Publication Date | n = 13 |
| Web of science (Clarivate) | May 2025 | TS=("data reuse" OR "reuse of data" OR "secondary data analysis" OR "secondary analysis")  AND TS=("health data" OR "clinical trial data" OR "patient data")  AND TS=("low and middle income countries" OR LMIC OR "global health" OR "low resource" OR LRS  OR "resource-poor setting" OR "resource-poor settings" OR "less developed countries"  OR "emerging economies" OR "under-resourced countries" OR "resource-constrained settings"  OR "low-GDP countries" OR "economically developing nations" OR "low- and middle-resource countries"  OR "global south") | Publication date: before 2022 | By Publication Date | n = 0 |

**PubMed search strategy**


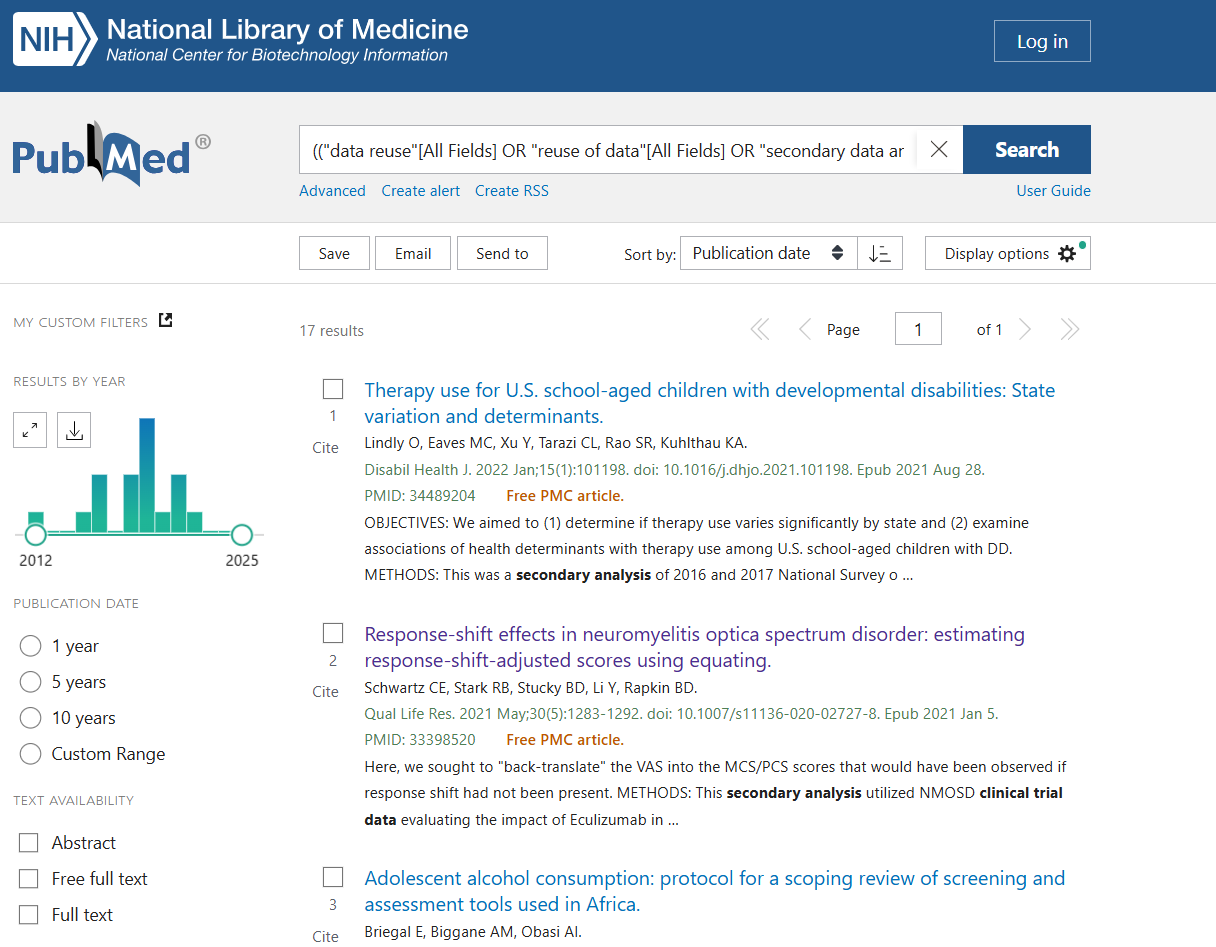


**SCOPUS search strategy**


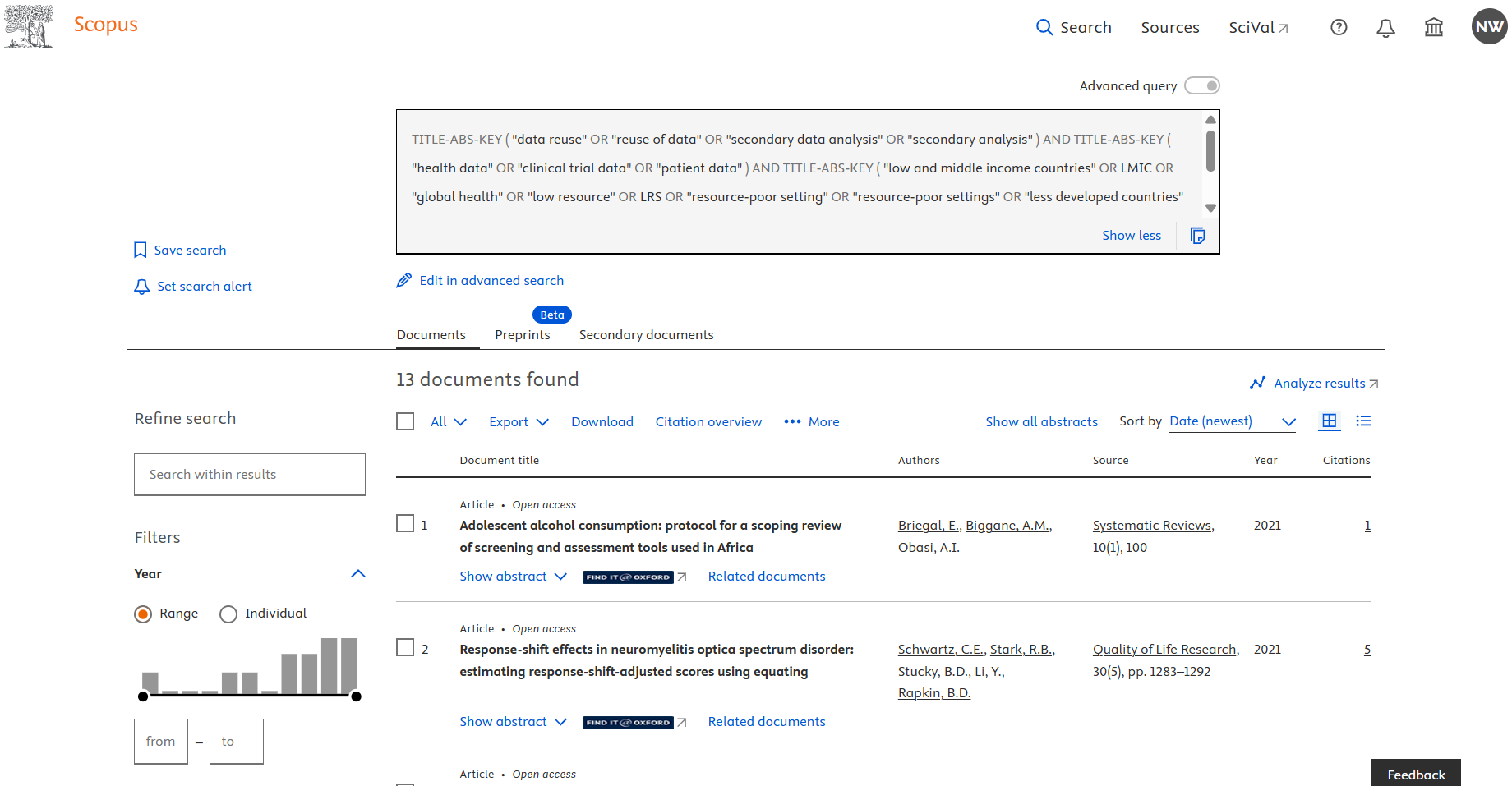


**Clarivate search strategy**


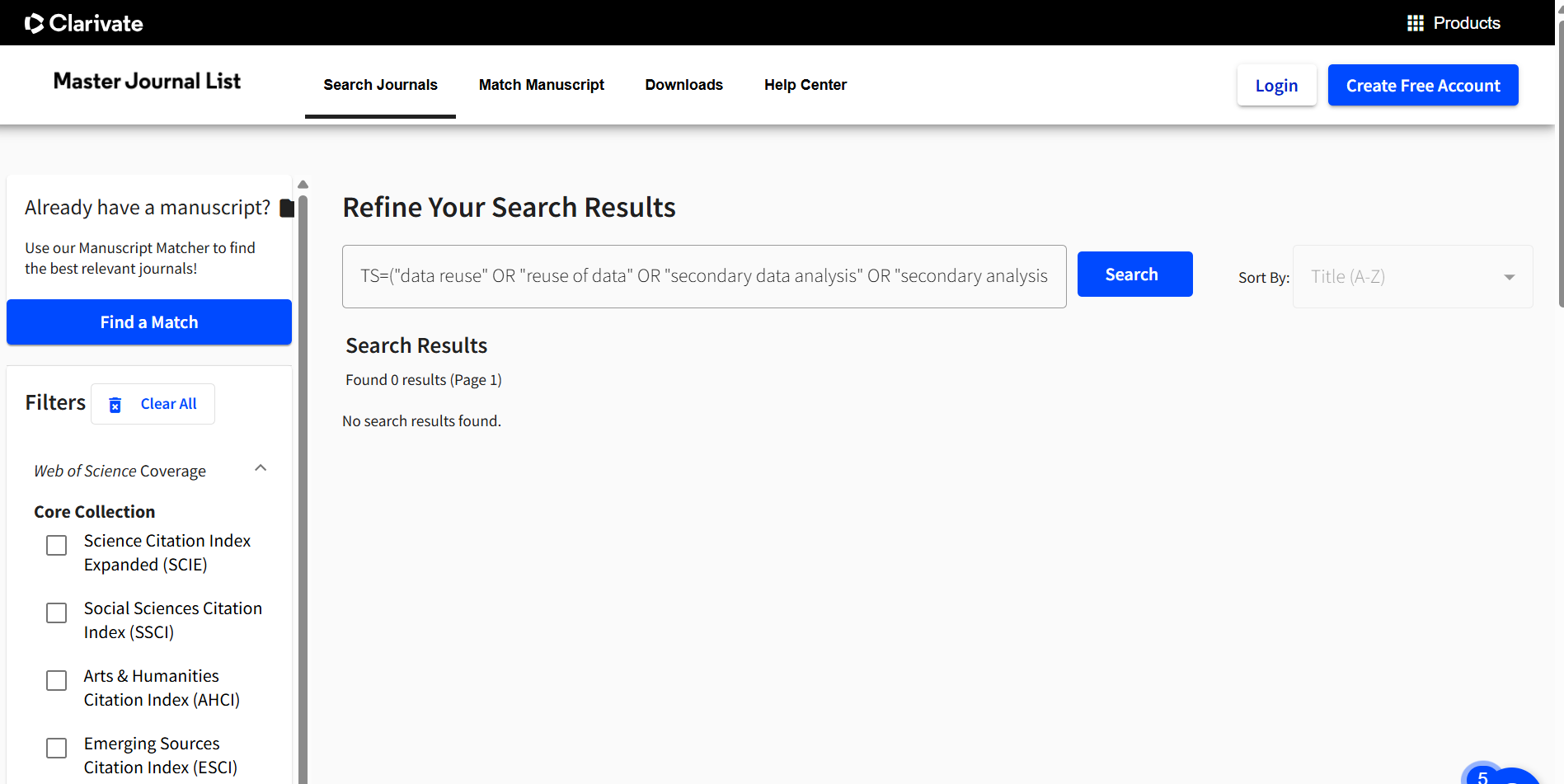

Supplement: online supplemental file 1 [file bmjgh-11-3-s001.docx]
