# Supplementary material for: Data reuse in global health: perspectives from actors in policy, funding and research
Source: BMJ Glob Health. 2026 Mar 4;11(3):e021974. doi: 10.1136/bmjgh-2025-021974 (PMC12970136; doi:10.1136/bmjgh-2025-021974)
Supplement: online supplemental file 4 [file bmjgh-11-3-s004.docx]

### BMJ Global Health Author Reflexivity Statement

Adapted from Morton, B., Vercueil, A., Masekela, R., Heinz, E., Reimer, L., Saleh, S., Kalinga, C., Seekles, M., Biccard, B., Chakaya, J., Abimbola, S., Obasi, A. and Oriyo, N. (2022), Consensus statement on measures to promote equitable authorship in the publication of research from international partnerships. Anaesthesia, 77: 264-276. <https://doi.org/10.1111/anae.15597>

| **Study conceptualisation** | |
| --- | --- |
| 1. How does this study address local research and policy priorities? | The study examines how data sharing translates into tangible benefits for research and health policy in low- and middle-income countries (LMICs) by analysing how shared datasets are used in practice and the factors that enable or constrain ethical and efficient secondary use. In doing so, it informs ongoing discussions on data governance, capacity, and responsible data use in LMIC settings. |
| 1. How were local researchers involved in study design? | Researchers based in Thailand, Vietnam, Kenya and Brazil were directly involved in designing the study protocol, and provided local contextual knowledge and research expertise. |
| **Research management** | |
| 1. How has funding been used to support the local research team(s)? | Funding was used to support training and mentorship and to enable dissemination of results, thereby increasing the visibility of locally led contributions. |
| **Data acquisition and analysis** | |
| 1. How are research staff who conducted data collection acknowledged? | All staff involved in data collection also contributed scientifically to the study and are thus included as authors. |
| 1. How have members of the research partnership been provided with access to study data? | Interview transcripts, codebooks, and draft manuscripts were shared with collaborators across participating units. |
| 1. How were data used to develop analytical skills within the partnership? | Partners strengthened analytical skills through hands-on analysis alongside experts in statistical and qualitative methods. |
| **Data interpretation** | |
| 1. How have research partners collaborated in interpreting study data? | Research partners jointly coded and analysed data across multiple sessions. |
| **Drafting and revising for intellectual content** | |
| 1. How were research partners supported to develop writing skills? | Through joint drafting, iterative feedback, and collaborative revision of the protocol, interview guides and manuscripts. |
| 1. How will research products be shared to address local needs? | Research outputs will be disseminated through open-access publication and on social media as appropriate. |
| **Authorship** | |
| 1. How is the leadership, contribution and ownership of this work by LMIC researchers recognised within the authorship? | Through inclusive authorship and explicit acknowledgement of author’s contribution. The first and last authors are LMIC researchers. |
| 1. How have early career researchers across the partnership been included within the authorship team? | Early career researchers were included as co-authors where they made meaningful contributions to data collection, analysis, or drafting, with senior researchers providing mentorship throughout the study. |
| 1. How has gender balance been addressed within the authorship? | Authorship was based on contribution, with gender representation across the author group. |
| **Training** | |
| 1. How has the project contributed to training of LMIC researchers? | Through hands-on involvement in mixed-methods research, including survey design, qualitative analysis, and collaborative interpretation of findings. |
| **Infrastructure** | |
| 1. How has the project contributed to improvements in local infrastructure? | While not focused on physical infrastructure, the project strengthened institutional capacity by reinforcing practices related to data governance, secondary data use, and collaborative research workflows. |
| **Governance** | |
| 1. What safeguarding procedures were used to protect local study participants and researchers? | The study was conducted under approved ethical protocols, with procedures in place to ensure informed consent, confidentiality, secure data handling, and protection of both participants and researchers. |
